# PHF8/KDM7B: A Versatile Histone Demethylase and Epigenetic Modifier in Nervous System Disease and Cancers

**DOI:** 10.3390/epigenomes8030036

**Published:** 2024-09-15

**Authors:** Tingyu Fan, Jianlian Xie, Guo Huang, Lili Li, Xi Zeng, Qian Tao

**Affiliations:** 1 Hunan Province Key Laboratory of Tumor Cellular & Molecular Pathology, Cancer Research Institute, Hengyang Medical School, University of South China, Hengyang 421001, China; tingyu@stu.usc.edu.cn (T.F.); huangguo@usc.edu.cn (G.H.); 2 Cancer Epigenetics Laboratory, Department of Clinical Oncology, State Key Laboratory of Translational Oncology, Sir YK Pao Center for Cancer, The Chinese University of Hong Kong, Hong Kong; jianlianxie@cuhk.edu.hk (J.X.); lili_li@cuhk.edu.hk (L.L.); 3 Department of Thyroid and Breast Surgery, The First Affiliated Hospital of Shenzhen University, Shenzhen Second People’s Hospital, Shenzhen 518035, China

**Keywords:** PHF8/KDM7B, epigenetics, lysine demethylation, malignant tumors, neurological diseases

## Abstract

Many human diseases, such as malignant tumors and neurological diseases, have a complex pathophysiological etiology, often accompanied by aberrant epigenetic changes including various histone modifications. Plant homologous domain finger protein 8 (PHF8), also known as lysine-specific demethylase 7B (KDM7B), is a critical histone lysine demethylase (KDM) playing an important role in epigenetic modification. Characterized by the zinc finger plant homology domain (PHD) and the Jumonji C (JmjC) domain, PHF8 preferentially binds to H3K4me3 and erases repressive methyl marks, including H3K9me1/2, H3K27me1, and H4K20me1. PHF8 is indispensable for developmental processes and the loss of PHF8 enzyme activity is linked to neurodevelopmental disorders. Moreover, increasing evidence shows that PHF8 is highly expressed in multiple tumors as an oncogenic factor. These findings indicate that studying the role of PHF8 will facilitate the development of novel therapeutic agents by the manipulation of PHF8 demethylation activity. Herein, we summarize the current knowledge of PHF8 about its structure and demethylation activity and its involvement in development and human diseases, with an emphasis on nervous system disorders and cancer. This review will update our understanding of PHF8 and promote the clinical transformation of its predictive and therapeutic value.

## 1. Introduction

Epigenetics is an intensively studied area referring to the alteration of gene activity and heritable phenotypes independent of DNA sequence changes [1]. Epigenetic reprogramming events are involved in a vast array of developmental processes and pathological states. Its modifications mainly encompass DNA CpG methylation, histone modifications, chromatin remodeling, and non-coding RNA regulation, which are fundamental to many biological processes, including transcriptional regulation, genomic imprinting, and X-chromosomal inactivation [2,3,4,5,6,7]. Histone is the condensed chromosomal core in the nucleus as the form of an octameric structure [8,9]. There are multiple amino acid residues on four core histones (H2A, H2B, H3, and H4) which can undergo various post-translational modifications, including methylation, acetylation, ubiquitination, and glycosylation, resulting in different biological functions [10,11]. Among them, methylation is a classic form of histone modification, with the functional outcomes determined by the methylation site and level [12]. On histone 3, methylation occurs on lysine (K) residues (K4, K9, K27, K36, K79) and arginine (R) residues (R2, R8, R17, R26). On histone 4, K20 and R3 are methylated. H3K4me3 and H3K79me3 are associated with active gene transcription, while H3K9me3, H3K27me3, H3K36me2/3, and H4K20me3 are repressive markers [13]. Numerous reports have pointed out that histone modifications contribute to the occurrence and development of cancer and neurological diseases and targeting drugs that intervene with histone modifications also demonstrate good clinical therapeutic potential [3,12,13,14].

The chromatin modification machinery includes three main members, namely a “writer”, “eraser”, and “reader” responsible for dynamic chromatin changes [14]. Histone methylation is reversibly orchestrated by histone methyltransferases (HMTs) and demethylases. Based on substrate specificity, HMTs are classified into two subfamilies, which are lysine specific methyltransferases (KMTs) and protein arginine-specific methyltransferases (PRMTs). To date, three types of histone demethylases have been identified that are responsible for removing methyl groups. These are peptidyl arginine deiminase 4 (PAD 4), flavin adenine dinucleotide (FAD)-dependent amino oxidase homolog lysine demethylase 1 (KDM1), and the Jumonji C (JmjC) domain-containing proteins [15,16,17,18]. JmjC domain-containing histone demethylases (JHDMs) belong to the 2-oxoglutarate oxygenase family, catalyzing lysine demethylation with iron Fe (II) and α-ketoglutarate (αKG) as cofactors in an oxidative reaction. Based on structural similarity, JHDMs are categorized into the following seven groups phylogenetically: JHDM1, PHF2/PHF8, JARID1/JARID2, JHDM3/JMJD2, UTX/UTY, JHDM2, and the JmjC domain-only family [19].

Plant homologous domain finger protein 8 (PHF8), also known as KDM7B, was identified as a chromatin regulator preferentially binding at promoters. Of note, mutations in PHF8 cause X-linked intellectual impairment (XLIR) and facial dysmorphism (cleft lip and palate) [20]. PHF8 overexpression is reported in several kinds of solid and hematologic malignancies, including cancer of the prostate, breast, gastric, liver, lung, and colon, together with acute promyelocytic leukemia (APL) and acute myeloid leukemia (AML) [21,22,23,24,25,26,27,28]. These observations show that PHF8 is a crucial protein for neurodevelopmental processes and malignant transformation through modulating the chromatin environment and gene transcription. Thus, this review was summarized to provide a rational foundation for future therapeutic development targeting PHF8, mainly focusing on its structure and demethylation activity and biological functions, as well as its involvement in development processes, with an emphasis on neurological disorders and cancer.

## 2. PHF8 Is a Histone Demethylase and an Epigenetic Modifier

### 2.1. Structure of PHF8

**Figure 1:**
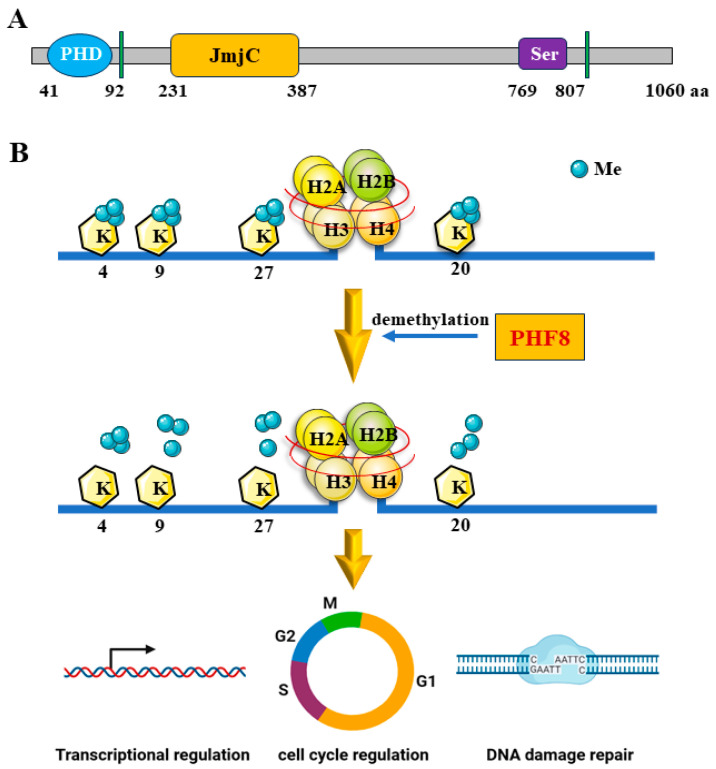
(**A**) Protein structure of PHF8. PHF8 domains are shown in different colors. In blue: plant homology domain (PHD); in green: nuclear localization signals; in orange: Jumonji C (JmjC) domain; in purple: serine-rich region (Ser). (**B**) Demethylation activity and biological functions of PHF8. Upper lane: histone lysine sites and number of methyl groups demethylated by PHF8; lower lane: fundamental cellular processes regulated by PHF8.

PHF8/KDM7B belongs to the KDM7 subfamily of the largest histone demethylase (KDM) family, the other two members of which are KDM7A/KIAA1718/JHDM1D and PHF2/KDM7C. PHF8 consists of 1060 amino acids and several domains, including a zinc finger plant homology domain (PHD), a JmjC domain, nuclear localization signals, and a serine-rich region (Figure 1A) [29]. Notably, the flexible PHD domain located at the N-terminus is equipped with recognition capability and the stable C-terminal JmjC domain performs catalytic functions. The PHD motif not only interacts with DNA to participate in transcriptional regulation, but also participates in histone substrate binding to influence post-translational modifications [30]. The catalytic domain has a high dioxygenase activity, which is dependent on a bivalent iron ion and 2-oxoglutaric acid and is closely associated with the presence of oxygen [31,32,33,34]. The structural mutations within its encoded JmjC domain and double-stranded β helix exon result in the loss of enzyme activity and gene dysfunction. In a crystal structure study, F279 was found in the enzyme activity center ring formed by the hydrophobic network. A point mutation at this site leads to damage to the enzyme activity center structure and the consequent loss of enzymatic activity of PHF8 [30]. Furthermore, the flexible linker located between the PHD domain and the JmjC domain is also critical for enzyme activity, as it promotes the better binding of sites.

### 2.2. Demethylation Activity of PHF8

PHF8, a nuclear protein, plays multiple epigenetic regulatory roles due to its histone methylation binding activity (as a reader) and demethylation activity (as an eraser) [35]. On one hand, PHF8 acts as a reader by preferentially binding to a specific histone modification site (H3K4me3), thus activating gene expression as a transcriptional activator [36]. On the other hand, the demethylase activity of PHF8 is relatively constant, with a specially structured hydrophobic core stabilizing its function [30]. The catalytic JmjC domain is responsible for removing the methyl groups from H3K9me2, which exerts an inhibitory effect on transcription [35]. Moreover, PHF8 facilitates gene regulation by removing the methyl groups from histone tails at specific sites, such as the well-known epigenetic repressive marks (H3K9me1/2, H3K27me2, and H4K20me1) (Figure 1B).

An analysis using calf histones as substrates showed that PHF8 selectively removes methyl groups from mono- and di-methylated sites, but does not accept tri-methylated substrates [37]. In contrast, Jumonji domain-containing protein 2A (JMJD2A)/KDM4A targets di- and tri-methylated sites [38]. This variation is linked to differences in the crystal structure of their catalytic domains. Interestingly, PHF8 was found to remove mono-methylation from lysine residues of DNA topoisomerase II-beta-binding protein 1 (TOPBP1), which are non-histone targets [39]. Thus, the PHF8 enzyme catalyzes the demethylation of specific histone residues, playing a role in the epigenetic regulation of gene expression.

### 2.3. Biological Functions of PHF8

The reversible dynamic methylation of histone lysine residues is a major contributor to genome stabilization, transcriptional regulation, and epigenetic effects [12,40]. Previous studies have shown that PHF8 is able to bind to more than one-third of all human genes [35,41]. However, depending on the cellular context, PHF8 controls the physiological functions of only about 2–5% of its direct target genes [35].

Through its demethylase activity, PHF8 has been implicated in a growing number of fundamental biological processes, including transcriptional regulation, cell cycle regulation, and DNA damage repair (DDR) (Figure 1B). As a transcriptional coactivator, PHF8 is primarily localized at the transcription start site (TSS) and acts on the following histone lysine sites: H3K9me1/2, H3K27me2, H4K20me1, and H3K36me2 [35,41], which upon the initiation of PHF8 demethylase activity cause varying degrees of histone demethylation and lead to PHF8-associated transcriptional regulation. Reported target genes of PHF8 will be discussed in Section 4.2.

The modulation of PHF8 on the cell cycle occurs through methylated histone lysine sites, resulting in the elimination of repressive markers to regulate the G1-S and G2-M phase transitions of the cell cycle. Specifically, PHF8 promotes S phase progression by binding to the cyclin E promoter and reducing H3K9me2 levels [42]. PHF8 also promotes the G1-S transition and dissociation from chromatin in early mitosis by removing the inhibitory H4K20me1 mark from promoters of transcription factor E2F1 *E2F1*-regulated genes [41]. In addition, as a G2/M regulator, PHF8 interacts with the CDC20-containing anaphase-promoting complex (APC) during mitosis and is subject to polyubiquitination-mediated degradation [43].

DNA is the carrier of genetic information, and when damaged causes severe harm to the organism [44]. There are two pathways for repairing DNA double-strand breaks (DSBs), homologous recombination (HR) and non-homologous end joining (NHEJ) repair [45,46]. Studies have shown that PHF8 is involved in DDR and acts in combination with ubiquitin-specific protease 7 (USP7), which stabilizes PHF8, to counteract genotoxicity in the DNA damage response. PHF8 homologs in C. elegans promote DNA repair through HR, whereas both NHEJ and HR repair for DSB in mammalian cells require the demethylase activity of PHF8 [45]. PHF8 is mobilized and recruited to DSB sites, promoting efficient DSB repair via HR or NHEJ pathways through the recruitment of BLM RecQ-like helicase (BLM) or KU70, respectively. Furthermore, the E3 ligase ring finger protein 168 (RNF168), a substrate of USP7, is part of the ubiquitin-dependent DNA damage signal and its expression decreases following USP7 deletion. However, the recruitment of DSB damage repair associated with PHF8 remains unaffected and altering PHF8 expression does not have any impact on the expression or function of RNF168 [47].

## 3. PHF8 in Development

### 3.1. PHF8 in Embryonic Development

The deletion of PHF8 ortholog 4F429 leads to embryonic death in C. elegans, suggesting its critical role in embryonic development [48]. In other species, although the absence of PHF8 does not completely prevent embryonic development, its presence is essential for maintaining the healthy and stable regulation of growth and development. The deletion of mouse *Phf8* results in a significant decrease in the proliferation rate of embryonic stem cells, neural precursor cells, and embryonic fibroblasts [49]. Moreover, knocking out the *Phf8* gene in mouse embryonic stem cells affects only the mesoderm, not the endoderm or ectoderm, which induces the stem cells to differentiate into cardiomyocytes [50].

### 3.2. PHF8 in Nervous System

**Figure 2:**
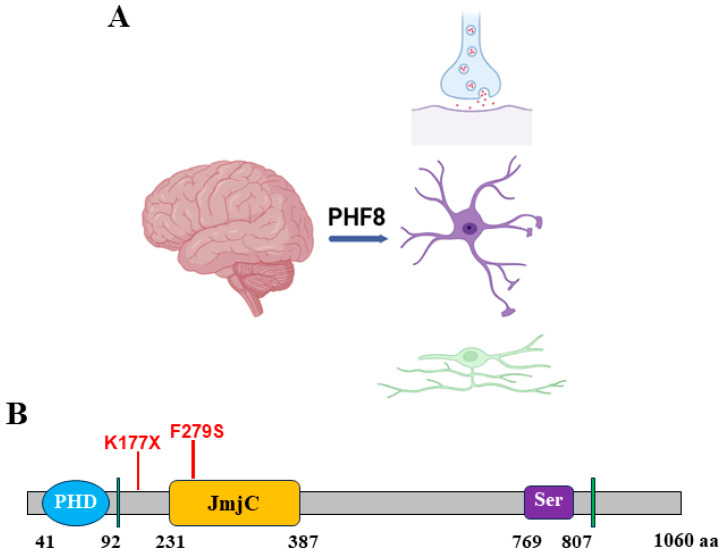
PHF8 in nervous system. (**A**) Role of PHF8 in nervous system. (**B**) Clinically observed PHF8 variants associated with X-linked intellectual disability.

Until now, studies of PHF8 in the nervous system have revealed its contribution to neuronal synapse formation, learning, and memory, with its variants causing neuropsychiatric disorders (Figure 2A). PHF8 expression is readily detected in brain structure sections of mouse embryos, particularly in the neocortex, midbrain, and dorsal medulla oblongata. Positive expression is also observed in the granular layer of the cerebellum and the hippocampus of adult mice, suggesting that PHF8 may be associated with learning and memory [51]. As reported, PHF8 specifically removes the inhibitory histone mark H3K9me2 from neurons and interacts with the acetyltransferase lysine acetyltransferase 5 (KAT5)/Tip60, promoting the formation of transcriptionally permissive phospho-acetylated histone H3K9ac-S10P (serine10), which has important implications for the epigenetic regulation of neuronal activity and the treatment of learning and memory disorders [52]. PHF8 plays a role in maintaining proper astrocyte development, mainly by stabilizing chromatin status and limiting the heterochromatin formation of presynaptic genes [53]. PHF8 promotes the proliferation rather than the differentiation of oligodendrocyte progenitors by upregulating the expression of oligodendrocyte transcription factor 2 (Olig2) [54]. The demethylation of H4K20me1 by PHF8 is attributed to the downregulation of cytoskeleton-related genes, and the dysregulated cytoskeleton organization can lead to abnormal neuronal connectivity and defective neurite outgrowth [55]. Additionally, neuronal differentiation is also regulated by retinoic acid receptors (RARs) and PHF8 acts as a co-activator of RARα [56]. The C. elegans homolog of PHF8, JMJD-1.2, controls proper axonal guidance by regulating Hedgehog-like signaling [57]. These findings demonstrate that PHF8 is strongly involved in neurodevelopmental processes and support the implication of PHF8 variants in nervous system diseases. 

The genes associated with cognitive function are typically located on the X chromosome, with mutations causing X-linked intellectual disability (XLID) [58]. Targeted next-generation sequencing has identified *PHF8* as a potential candidate gene [59]. Further gene linkage analysis exhibits that the absence of PHF8 function leads to cognitive impairment and developmental abnormalities specific to the X chromosome among human beings. Several cases of cognitive abnormalities and craniofacial deformities due to *PHF8* genetic variations have been reported (Figure 2B). First, the original family of Siderius–Hamel CL/P syndrome presents a nonsense mutation (p.K177X) wherein patients exhibit varying degrees of intellectual disability and a cleft lip and palate. Gene sequencing reveals the replacement of 529 base T at exon 6 of the *PHF8* gene with A (c.529A > T). The premature termination of protein translation leads to the loss of the JmjC domain and five nuclear localization signals in the PHF8 protein [60,61]. Second, a recent discovery of a *PHF8* missense mutation on exon 8, comprising a shift from the hydrophobic phenylalanine within the JmjC domain to a more polar and hydrophilic serine (F279S), has been found within a Finnish family of male patients displaying mild cognitive impairment alongside clinical features of a cleft lip and palate [62]. The regular nuclear location of PHF8 is changed in F297S mutants to a different cytoplasmic location, leading to the loss of its demethylase activity. Extensive facial deformities resulting from disruptions of *PHF8* coding worsen with age. Adverse concomitants, such as speech and developmental delays, psychomotor disorders, sensory abnormalities, autism, attention-deficit hyperactivity disorder (ADHD), anxiety, and violent behavior were observed in 11 variants and 16 individuals affected by the predictive loss of PHF8 function [60]. It was reported that a predicted mutation caused amino acid frame deletion of the 969 serine of PHF8 [63], and unidentified but PHF8-associated mutations potentially affect chromatin regulation. Patients exhibiting symptoms comparable to PHF8–XLID are prevalent among siblings, frequently with congenital abnormalities and heritable traits [60]. PHF8 recruits XLID-related genes *JARID1C* and *ZNF711* around the H3K4me3-positive regions at the TSS sites. These proteins interact with one another and are functionally linked, leading to a complex clinical phenotype [64]. Intriguingly, mutations of JARID1C within the finger domain of JmjC and PHD have also been detected in X-linked intellectual disability (XLID) cases [65]. JARID1C and PHF8 are part of a vast gene family of histone lysine-specific demethylases (LSDs), also known as lysine demethylases (KDMs). This infers the role of this gene family in intellectual development, deserving further investigation.

The effect of PHF8 loss has also been studied in mice and non-specific forms of XLID have been observed. Impaired learning and memory and hippocampal long-term potentiation were found in *Phf8* knockout mice, but without obvious morphological defects [66]. However, instead of developmental deficits and cognitive impairments, anxiety and depression-like behaviors were observed in *Phf8* deficient mice, which is thought to be mediated by the direct regulation of serotonin receptors (5-Hydroxytryptamine receptor 1a/2a (Htr1a, Htr2a) [67]. Such divergence may be due to the complexity of the genetic background. Also, the degree of abnormal symptoms varies among carriers of PHF8 variants, suggesting the involvement of other regulators or environmental factors.

### 3.3. PHF8 in Other Systems

In addition to the nervous system, PHF8 is involved in various biological and pathological processes in other systems. PHF8 has been reported to regulate osteogenic differentiation *via* Wnt/β-catenin signaling in a rat osteoporosis model, showing potential value as a therapeutic target [68]. In the cardiovascular system, PHF8 is under-expressed in cardiac mast cells during myocardial pathology and can reverse hypertrophy caused by an excessive cardiac workload in cardiomyocytes [69]. Specifically, cardiac-specific PHF8 overexpression reduces AKT and mTOR phosphorylation induced by abdominal aortic coarctation, and it inhibits perivascular and interstitial fibrosis, termed cardiac fibrosis. However, the histone demethylase KDM3C, which indirectly inhibits PHF8 transcription, relies on epigenetic modifications to promote cardiac hypertrophy [70]. In zebrafish, the transcriptional regulation of PHF8 is required for the development of the retro-auricular auditory organs. PHF8 inhibition by morphine reduced inner ear hair cell differentiation and the number of lateral nerve mast cells [71].

## 4. PHF8 in Cancer

Genomic instability is a hallmark of cancer [72]. The alterations and functional roles of PHF8 have been extensively studied in multiple solid tumors and hematological malignancies, serving as an oncogenic factor. Current studies of PHF8 in cancer are summarized in Table 1, including its expression abnormalities, biological functions, and underlying mechanisms. During malignant transformation, PHF8 acts as a hub of the complex cellular network regulated by diverse factors, and it relays information to many downstream effectors, triggering signaling cascades and phenotype alteration.

### 4.1. Regulation of PHF8 in Tumorigenesis 

As reported, PHF8 is regulated at three levels, namely the transcriptional, post-transcriptional, and post-translational levels. First, PHF8 is under the control of hypoxia in clear-cell renal carcinoma (ccRCC). Hypoxia-inducible factors (HIFs) (HIF1α, HIF2α) are recruited to the *PHF8* promoter and activate its transcription, which explains the lipid deposition induced by von Hippel–Lindau (VHL) deficiency [87]. HIF1α and HIF2α-dependent PHF8 regulation is also observed in castration-resistant prostate cancer (CRPC). PHF8 elevation was abolished in HIF1α and HIF2α knockdown prostate cancer cells. A higher PHF8 expression is observed in CRPC patients with an advanced grade and poor survival. HIFs promote androgen receptor (AR) activation and PHF8 functions as a connecting bridge between AR and HIFs. This HIFs/AR/PHF8 axis accelerates prostate cancer progression and is a potential therapeutic target for CRPC treatment [88]. In turn, PHF8 is essential for HIF1α activation and the induction of its target genes by maintaining the active mark H3K4me3 [89]. The interplay between PHF8 and HIFs may shed light on hypoxia-induced neuroendocrine differentiation (NED) and resistance to androgen deprivation therapies in prostate cancer.

**Figure 3:**
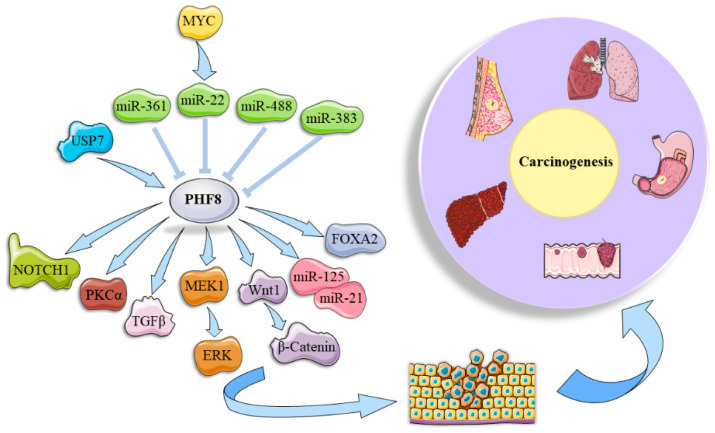
Regulators and downstream effectors of PHF8 in tumorigenesis.

Post-transcriptionally, microRNAs are the main regulators of PHF8 expression (Figure 3). A MYC/miR-22-3p/PHF8 regulatory axis has been identified in gastric cancer. MYC increases PHF8 protein levels rather than mRNA expression via stabilizing miR-22-3p, promoting the proliferation and migration or invasion of gastric cancer cells [76]. Consistently, PHF8 was found to be a direct target of miR-22 and is thus mediated by MYC to promote the epithelial–mesenchymal transition (EMT) and tumorigenesis in breast cancer [75]. In prostate cancer, miR-22 can cause partial NED and directly inhibit PHF8 translation by targeting the 3′-untranslated region (3′-UTR). This axis is a downstream effector of AR signaling and leads to the proliferation of CRPC cells [73]. Moreover, there are other microRNAs that target and regulate the expression of PHF8. MiR-488, a tumor suppressor [90], targets the 3′-UTR of *PHF8* mRNA in colorectal cancer and inhibits its expression to suppress the growth and metastasis of colorectal cancer [78]. MiR-383 can reduce the *PHF8* mRNA level to inhibit the proliferation, migration, and invasion of liver cancer cells [79]. Oncogenic lncRNA BBOX1-AS1 acts as a competing endogenous RNA (ceRNA), contributing to liver cancer progression. Sponge adsorption of miR-361-3p drives the expression of PHF8 in liver cancer to promote cancer progression and regulate drug sensitivity to sorafenib via autophagy [80].

Post-translationally, the protein level and function status of PHF8 are modified by phosphorylation and ubiquitination. All-trans retinoic acid (ATRA) is administered as a drug to combat APL. PHF8 resurrects the sensitivity of APL cells to ATRA treatment, dependent on its serine phosphorylation (S33, S84) and enzymatic activity [27]. Furthermore, the induction of PHF8 site-specific phosphorylation by ATRA exposure is in a dose-dependent manner. Phospho-PHF8 upregulates cytosolic RNA sensors and confers apoptosis mediated by the IFN-I response. These findings provide a novel insight to overcome ATRA resistance and enhance the anti-leukemic activity of PHF8 by the pharmacological induction of its phosphorylation [28]. PHF8 is also subjected to degradation via the ubiquitin–proteasome system. One study shows that NEDD4L (E3 ubiquitin–protein ligase neural precursor cell expressed developmentally downregulated 4-like) interacts with PHF8 and induces its degradation by ubiquitination. Decreased PHF8 leads to the enrichment of H3K9me2 in the promotor region of activating transcription factor 2 (*ATF2*) and permits its transcription, thereby inhibiting the proliferation of prostate cancer cells [79]. PHF8 is a substrate of USP7. The C-terminal domain of PHF8 interacts with the meprin and TNF receptor-associated factor (TRAF) homology (MATH) domain on the N-terminus of USP7. The two components work in tandem to govern the expression of cell cycle factors, such as cyclin A2, to encourage the proliferation of breast cancer cells [22].

In addition to cellular molecules, PHF8 expression is also affected by environmental factors. In vitro and in vivo studies provide evidence of Helicobacter pylori’s ability to elicit PHF8 expression during gastric cancer progression [77]. Coincidentally, the indirect pathogenic mechanism of H. pylori infection can also be applied to the JMJD2B-mediated progression of chronic gastritis to gastric cancer [91]. It is believed that nicotine in tobacco may also induce PHF8 overexpression in lung cancer. Epigenetic changes, oxidative stress responses, and inflammation induced by cigarette smoke have been validated in cross-over reviews of multiple omics [92]. The transcription factor PHF8 was identified as an interacting factor of smoking-related lung cancer using bioinformatics analysis and modeling [83].

### 4.2. Downstream Effectors of PHF8 and Involved Signaling Pathways

PHF8 exerts its histone demethylase effect on epigenetic inhibition by erasing methyl groups and has differential effects on different downstream effectors (Figure 3). First, some microRNAs serve as targets of PHF8 for its oncogenic roles in tumorigenesis. For example, PHF8 upregulates miR-125b expression to participate in the regulation of the proliferation and apoptosis of prostate cancer cells [74]. PHF8 promotes the proliferation and apoptosis of non-small cell lung cancer (NSCLC) by promoting the expression of miR-21, an oncogenic factor enhancing the growth, migration and invasion, and resistance to chemotherapy and radiotherapy by targeting phosphatase and tensin homolog (PTEN) [25,93].

By binding with H3K4me3, an active mark usually located near TSS, PHF8 is recruited and enriched in gene promoters and transcriptionally regulates their expression. PHF8 upregulates Forkhead Box A2 (*FOXA2*) expression by removing inhibitory histone markers in the *FOXA2* promoter, contributing to the progression of neuroendocrine prostate cancer [21]. PHF8 induces EMT-like cell status through upregulating key EMT transcription factors *SNAI1* and *ZEB1*, thus contributing to cancer cell growth and metastasis [75]. It was found that the presence of FIP200 is a prerequisite for autophagosome formation and autophagy in liver cancer [94]. PHF8 promotes E-cadherin degradation by accelerating autophagy and promotes the transcription of *FIP200*, *SNAI1*, and *VIM* by binding to their promoters. Also, PHF8 functions as an oncogene during liver cancer pathogenesis through increasing the expression of Cullin 4A (CUL4A) [81], a regulator of EMT.

PHF8 facilitates the proliferation and spreading of NSCLC via inducing *Wnt1* promoter activity and triggering Wnt/β-catenin signaling [82]. PHF8 also physically interacts with β-Catenin and is recruited to the mesenchymal marker *VIM* promoter to co-activate its transcription in gastric cancer cells [77]. In addition, PHF8 directly binds to the *MEK1* promoter, leading to *MEK1* transcriptional upregulation and the activation of the MER/ERK signaling pathway that promotes the proliferation of acute lymphoblastic leukemia, with the use of pathway inhibitor PD184352 potentiating the inhibitory effect of *PHF8* gene knockout [84]. PHF8 is involved in *HER2* transcriptional regulation via the maintenance of H3K4me3 levels, which is required for *HER2* transcriptional regulation in breast cancer cells [95]. In line with this, PHF8 directly regulates transcription factor AP-2 gamma (*TFAP2C*), a transcription factor that positively regulates *HER2*, thus promoting *HER2* expression and working synergistically with HER2 signaling [96]. Moreover, PHF8 promotes the migration and invasion of HER2-negative gastric cancer. A high expression of PHF8 is associated with an advanced tumor stage and inferior survival in gastric cancer patients. This is explained by PHF8 interaction with c-Jun and removal of H3K9me2 methylation to activate PRKCA (coding PKCα) and promote SRC-induced degradation of PTEN [23], a tumor suppressor commonly mutated in tumors and whose dysfunction is key to activate PI3K–AKT signaling. Highly expressed PHF8 expression in metastatic melanoma cells is crucial to the activation of the oncogenic pathway and the invasion of melanoma cells through its demethylase activity. PHF8 mutants lacking enzyme activity are unable to ameliorate the invasion defect brought on by *PHF8* knockout [86]. TGF-β signaling has a strong and effective induction effect on EMT, which is facilitated by PHF8’s gain-of-function in the context of TGF-β [75,97]. Although TGF-β has different roles in normal and tumor cells, PHF8 dysregulation contributes to the functional transformation of TGF-β from a cell suppressor to a pro-cancer agent in cancer cells [75]. Collectively, PHF8 transcriptionally regulates multiple target genes and facilitates the activation of oncogenic signaling networks, including Wnt/β-catenin, MER/ERK, PI3K–AKT, and TGF-β signaling, thereby aiding in cancer initiation and progression.

### 4.3. PHF8 and Tumor Immunity

PHF8 has also been implicated in tumor immune responses and its association with interleukin-6 (IL-6) has been validated in a number of cancer types [96]. Within the tumor microenvironment, IL-6 facilitates the infiltration of inflammation-induced CD8^+^ T cells [98,99] and cell-based models of NED and CRPC have been induced by IL-6 treatment in which PHF8 was found to be downregulated during NED and upregulated in CRPC [73]. In HER2-positive breast cancer, PHF8 influences the tumor microenvironment by regulating cytokine IL-6 production and promoting T cell migration into the tumor. In HER2-positive breast cancer mouse models, there are significantly fewer invasive CD4^+^ and CD8^+^ T cells in PHF8 knockout tumors [96]. Interference with PHF8 can cause the degradation of intracellular methyltransferase SETDB1 (SET domain bifurcated histone lysine methyltransferase 1), leading to the transcriptional activation of H3K9me3-modified retrotransposons, which will induce an intracellular antiviral response and an anti-tumor immune response [26].

## 5. Clinical Implications of PHF8 and Perspectives

As summarized above, the activity of the PHF8 enzyme plays a crucial role in the cognitive and intellectual development of children. Additionally, the mutation of the *PHF8* gene on the X chromosome is a heritable variation. This suggests that preconception genetic testing and screening for *PHF8* mutations in families with a history of XLIDmay be necessary to prevent possible risks. Furthermore, PHF8 may also be regulated by protein degradation. Research into the causes of variations within the *PHF8* gene can aid in predicting the severity of the disease, evaluating the growth and development of children, and offering potential interpretations of PHF8 variants of unknown significance (VUS) [60]. Mutations in the *PHF8* gene prompt changes in the physical structure of the enzyme active center, opening new avenues for mechanism exploration and disease prevention. Owing to its role in the development of auditory organs, PHF8 shows potential value for the treatment of hearing damage [71]. PHF8 reverses cardiac dysfunction, indicating that PHF8 could potentially aid in managing hypertrophic cardiomyopathy, which may have significant implications in preventing congestive heart failure and sudden cardiac death.

The diverse involvement of PHF8 in pathologic disorders, especially nervous system diseases and cancer, prompts us to evaluate its candidacy as a therapeutic target through the manipulation of its demethylase activity. Structure–function studies permit the development of inhibitors pharmacologically targeting PHF8. Pyridine derivatives represent one of the main areas for the development of JmjC protein inhibitors. A new pyridine derivative, which introduces particular substitutions to regulate the selectivity of JmjC enzyme family inhibitors, has been granted a patent US20160102096A1, but its bioavailability is currently unknown [100]. Compound 9, a KDM inhibitor for PHF8, KDM2A, and KDM7A, has been created. In an enzyme assay, it displays the most potent inhibitory effect on PHF8 and has been proven to hinder the proliferative activity of HeLa and KYSE150 cells. In addition, this inhibitor can also downregulate E2F1 expression, which is a transcription factor accelerating cell cycle progression. The prolongation of the G0/G1 phase of these two cell lines has indirectly verified the regulatory effect of PHF8 on the cell cycle [101]. Recently, based on the structure of the PHF8 catalytic core, a small-molecular inhibitor of PHF8 (iPHF8) was identified. iPHF8 exhibits potent efficacy in the regulation of the transcription of electron transport chain genes, the production of mitochondrial reactive oxygen species, and cell growth in colon and lung cancer cells, providing a promising tool for combating cancer [32].

In cancer management, chemotherapy and radiation therapy destroy tumor cells by inducing DNA damage. The damage is irreversible, that is, it cannot be repaired by the DDR mechanism [102]. However, cunning cancer cells can sometimes develop abnormal DDR mechanisms, making them resistant to DNA damage therapy [103], which is undoubtedly a potential danger for poor prognosis and treatment relapse. Based on this situation, the involvement of PHF8 in DSB repair has become a novel treatment idea in combination with chemoradiotherapy. For instance, both USP7 and PHF8 exhibit high expression in breast cancer and DSB repair relies on PHF8 stabilization, which is enforced by USP7. Thus, exploring the potential of the selective inhibition of USP7 and/or PHF8 enzyme activity, particularly in combination with chemotherapy or radiation therapy, may be worthwhile for treatment refinement of breast cancer. Certain scholars have discovered that PHF8 inhibitors may be used as synergistic reagents for HER2-positive breast cancer patients to overcome resistance to anti-HER2 therapies [96].

PHF8 inhibitors are attractive anticancer agents, either as monotherapy or in combination with other treatments; however, challenges remain in their identification and development. First, high specificity exclusively targets PHF8, rather than the other two subfamily members KDM7A and PHF2, to avoid functional redundancy. Second, the cellular activity of compounds designed based on structure modeling needs to be tested and in accordance with expectations. Third, how PHF8 regulates chromatic dynamics, except for transcriptional regulation, and the expression and function of PHF8 in cancer patients require further elucidation. Further investigations on PHF8 will be beneficial to overcome these obstacles and provide useful therapeutic approaches.

## 6. Conclusions

With PHD and JmjC domains, PHF8 protein binds to H3K4me3 and colocalizes with H3K4me3 at transcription start sites. PHF8 erases the repressive histone methyl marks, including H3K9me1/2, H3K27me1, and H4K20me1, and acts as a transcriptional activator of multiple target genes. PHF8 is indispensable for developmental processes, as emphasized by the association of PHF8 variants with intellectual disabilities and craniofacial dysmorphism. The oncogenic role of PHF8 has been reported in multiple cancers, wherein PHF8 involves various regulators, downstream effectors, and signaling cascades. Structure-based identification and development of PHF8 inhibitors are emerging as innovative approaches to combat human diseases.

## Figures and Tables

**Table 1:** The roles and mechanisms of PHF8 in different tumors.

Tumors	PHF8 Expression	Related Pathways and Acting Factors	Other Influencing Factors	Biological Function	Refs
Prostate cancer	High	MYC/miR-22/PHF8 PHF8/miR-125b PHF8/FOXA2	Hypoxia	Proliferation (+) Apoptosis (−)	[21,73,74]
Breast cancer	High	MYC/miR-22/PHF8 USP7/PHF8/Cyclin A2		Proliferation (+) EMT (+)	[22,75]
Gastric cancer	High	MYC/miR-22/PHF8 PHF8/β-catenin/Vimentin PHF8/PKCα/Src/PTEN	Helicobacter pylori	Proliferation (+) Migration (+) Invasion (+) EMT (+)	[23,76,77]
Colorectal cancer	High	miR-488/PHF8		Proliferation (+) Migration (+) Invasion (+)	[78]
Liver cancer	High	PHF8/CUL4A miR-383/PHF8 BBOX1-AS1/miR-361-3p/PHF8		Proliferation (+) Migration (+) Invasion (+) EMT (+) Drug resistance (+)	[79,80,81]
Lung cancer	High	PHF8/miR-21/PTEN PHF8/Wnt1/β-catenin	Nicotine	Proliferation (+) Migration (+) Invasion (+) Apoptosis (−) Drug resistance (+)	[25,82,83]
Acute lymphocytic leukemia	High	PHF8/MEK1/ERK PHF8/NOTCH1		Proliferation (+)	[84,85]
Metastatic melanoma	High	PHF8/TGFβ		Invasion (+)	[86]

## Data Availability

Not applicable.
